# Structural and Psychosocial Syndemic Conditions and Condomless Anal Intercourse Among Transgender Women — National HIV Behavioral Surveillance Among Transgender Women, Seven Urban Areas, United States, 2019–2020

**DOI:** 10.15585/mmwr.su7301a3

**Published:** 2024-01-25

**Authors:** Rebecca B. Hershow, Lindsay Trujillo, Evelyn Olansky, Kathryn Lee, Christine Agnew-Brune, Cyprian Wejnert, Monica Adams, National HIV Behavioral Surveillance Among Transgender Women Study Group, National HIV Behavioral Surveillance Among Transgender Women Study Group, Narquis Barak, Kathleen A. Brady, Sarah Braunstein, Jasmine Davis, Sara Glick, Andrea Harrington, Jasmine Lopez, Yingbo Ma, Aleks Martin, Genetha Mustaafaa, Tanner Nassau, Gia Olaes, Jennifer Reuer, Alexis Rivera, William T. Robinson, Ekow Kwa Sey, Sofia Sicro, Brittany Taylor, Dillon Trujillo, Erin Wilson, Pascale Wortley

**Affiliations:** ^1^Division of HIV Prevention, National Center for HIV, Viral Hepatitis, STD, and TB Prevention, CDC, Atlanta, Georgia; ^2^Social & Scientific Systems, Inc., Silver Spring, Maryland; CrescentCare; Philadelphia Department of Public Health; New York City Department of Health and Mental Hygiene; CrescentCare; University of Washington, School of Medicine, Division of Allergy and Infectious Diseases, Public Health – Seattle & King County, HIV/STD Program; Philadelphia Department of Public Health; New York City Department of Health and Mental Hygiene; Los Angeles County Department of Public Health; Public Health – Seattle & King County, HIV/STD Program; Georgia Department of Public Health; Philadelphia Department of Public Health; Los Angeles County Department of Public Health; Washington State Department of Health; New York City Department of Health and Mental Hygiene; Louisiana State University Health Science Center in New Orleans – School of Public Health, Louisiana Office of Public Health STD/HIV/Hepatitis Program; Los Angeles County Department of Public Health; San Francisco Department of Public Health; Georgia Department of Public Health; San Francisco Department of Public Health; San Francisco Department of Public Health; Georgia Department of Public Health

## Abstract

Psychosocial and structural syndemic conditions, including polydrug use and experiencing homelessness, frequently co-occur and might jointly increase HIV risk. Limited studies have assessed racial and ethnic differences in exposure to syndemic conditions and behaviors associated with HIV transmission among transgender women. This report examines the relation between syndemic conditions and condomless anal intercourse (CAI) among transgender women in seven urban areas in the United States to develop HIV prevention interventions for transgender women. During 2019–2020, transgender women in seven urban areas were recruited using respondent-driven sampling for a biobehavioral survey. Reported syndemic conditions (psychosocial: polydrug use, sexual violence, and psychological distress; structural: homelessness, incarceration, and exchange sex) were summed to create a syndemic score. Using modified Poisson regression to account for RDS, the study assessed whether the strength of the association between syndemic score and CAI differed by race and ethnicity. To assess additive interaction, the relative excess prevalence owing to interaction (REPI) and 95% CIs for selected pairs of syndemic conditions on CAI prevalence stratified by race and ethnicity were estimated. Of 1,348 transgender women (Black = 546, White = 176, and Hispanic = 626), 55% reported CAI; and 24% reported ≥3 syndemic conditions. Reporting additional syndemic conditions was associated with CAI for White, Hispanic, and Black participants. The association was significantly stronger for White than Black and Hispanic participants. Limited significant superadditive interactions were found, although the majority were between structural syndemic conditions. Racial and ethnic differences in REPI estimates were observed. Reporting more syndemic conditions was associated with increased CAI across racial and ethnic groups, demonstrating that HIV prevention efforts for transgender women should address structural and psychosocial syndemic conditions. Results differed by race and ethnicity, indicating that syndemic-focused interventions for transgender women should be tailored to racial and ethnic groups.

## Introduction

Transgender women are disproportionately affected by HIV, and severe racial and ethnic disparities in HIV prevalence among transgender women exist (*1*,*2*). Transgender women might be disproportionately affected by HIV because they experience high levels of social, legal, and economic marginalization, thereby increasing exposure to syndemic conditions, including experiencing homelessness, incarceration, exchange sex, polydrug use, violence, and psychological distress (*3*–*13*). Syndemic theory posits that epidemics are produced by both diseases and social conditions (*14*,*15*). The theory emphasizes how structural factors (e.g., experiencing homelessness and incarceration) and psychosocial factors (e.g., sexual violence and polydrug use) jointly increase risk for HIV acquisition and transmission (*14*,*15*). Differences in exposure to syndemic conditions by race and ethnicity might explain the racial and ethnic disparities in HIV prevalence (*7*). No studies have assessed racial and ethnic disparities in syndemic conditions and behaviors associated with HIV transmission in a population-based sample of transgender women (*16*).

This report examines the relation between structural and psychosocial syndemic conditions (experiencing homelessness, incarceration, exchange sex, polydrug use, sexual violence, and psychological distress) and condomless anal intercourse (CAI) among Black or African American (Black), White, and Hispanic or Latina (Hispanic), transgender women in the United States. (Persons of Hispanic origin might be of any race but are categorized as Hispanic; all racial groups are non-Hispanic.) These findings can be used to develop HIV prevention interventions tailored for racial and ethnic groups of transgender women.

## Methods

### Data Source

This report includes survey data from the National HIV Behavioral Surveillance Among Transgender Women (NHBS-Trans), which was conducted by CDC during June 2019–February 2020 to assess behavioral risk factors, prevention usage, and HIV prevalence. Eligible participants completed an interviewer-administered questionnaire and were offered an HIV test. Definitions of demographics and social determinants of health are available in the Overview and Methodology Report of this supplement (*17*). The NHBS-Trans protocol, questionnaire, and documentation are available at https://www.cdc.gov/hiv/statistics/systems/nhbs/methods-questionnaires.html#trans. This activity was reviewed by CDC, deemed not research, and was conducted consistent with applicable Federal law and CDC policy.*

Applicable local institutional review boards in each participating project area approved NHBS-Trans activities. The final NHBS-Trans sample included 1,608 transgender women in seven urban areas in the United States (Atlanta, Georgia; Los Angeles, California; New Orleans, Louisiana; New York City, New York; Philadelphia, Pennsylvania; San Francisco, California; and Seattle, Washington) recruited using respondent-driven sampling. This analysis is limited to 1,348 eligible participants who had an HIV-negative or HIV-positive National HIV Behavioral Surveillance (NHBS) HIV test result; identified as Black, White, or Hispanic; and had no missing outcome data.

### Measures

The outcome variable was past-year CAI, which was defined as having insertive or receptive anal sex without a condom during the past 12 months (Table 1). Psychosocial syndemic conditions included past-year polydrug use, past-year experience of sexual violence, and past-month psychological distress. Polydrug use was defined as having used speedball (combination of heroin and cocaine) or two or more types of drugs via injection or noninjection that were not provided by a health care professional during the past 12 months, including heroin, powder cocaine, crack cocaine, methamphetamine, painkillers (e.g., Oxycontin, Vicodin, morphine, or Percocet), downers (e.g., Klonopin, Valium, Ativan, or Xanax), or poppers or amyl nitrate; marijuana, alcohol, and fentanyl were not included. Experience of sexual violence was defined as being physically forced or verbally threatened to have sex when they did not want to during the past 12 months. To measure psychological distress, participants completed the validated, widely-used Kessler Psychological Distress Scale comprising six items asking participants how often they have been feeling emotions (e.g., nervous or hopeless) during the past 30 days; response options ranged from “All of the time” to “None of the time” (*18*–*20*). Participants with a composite score of 13–24 were categorized as experiencing psychological distress; those with a score of <13 were categorized as not experiencing psychological distress (*18*,*19*).

**TABLE 1:** Variables, questions, and analytic coding for selected sociodemographic characteristics, syndemic conditions, and occurrence of condomless anal intercourse among transgender women — National HIV Behavioral Surveillance System Among Transgender Women, seven urban areas,* United States, 2019–2020

Variable	Question	Analytic coding
**Sociodemographic characteristic**		
Age at interview, yrs	What is your date of birth?	18–24, 25–29, 30–39, 40–49, or ≥50
Education	What is the highest level of education you completed?	<High school, high school diploma or equivalent, some college or technical degree, or college degree or more
Relationship status	Of the [total number] sex partners you’ve had in the past 12 months, how many would you consider main partners? By main partner, I mean a person you have sex with and who you feel committed to above anyone else. This is a partner you would call your boyfriend, girlfriend, significant other, or life partner.	Partnered (reported having at least one main sexual partner) or single (reported having no main sexual partners)
Health insurance	Do you currently have health insurance or health care coverage?	Yes or no
NHBS HIV test result	NA^†^	HIV positive or HIV negative
Race and ethnicity^§^	Do you consider yourself to be of Hispanic, Latino/a, or Spanish origin? Which racial group or groups do you consider yourself to be in? You may choose more than one option.	Non-Hispanic Black or African American, Non-Hispanic White, or Hispanic or Latina
Urban area	NA^¶^	Atlanta, GA; Los Angeles, CA; New Orleans, LA; New York City, NY; Philadelphia, PA; San Francisco, CA; or Seattle, WA
**Psychosocial syndemic condition**		
Polydrug use	*Injection drug use questions:* In the past 12 months, which drug did you inject most often? What other drugs did you inject? *Non-injection drug use questions:* In the past 12 months, did you use any of the following drugs? Methamphetamine (including meth, crystal, speed, or crank)? Crack cocaine? Powder cocaine that is smoked or snorted? Downers (benzos) such as Klonopin, Valium, Ativan, or Xanax? Painkillers such as Oxycontin, Vicodin, morphine, or Percocet? Heroin that is smoked or snorted? Poppers or amyl nitrate?	Yes (reported having used speedball [combination of heroin and cocaine] or two or more of the following types of drugs via injection or non-injection: heroin, powder cocaine, crack cocaine, methamphetamine, painkillers such as Oxycontin, Vicodin, morphine, or Percocet, downers such as Klonopin, Valium, Ativan, Xanax, or poppers or amyl nitrate) or no
Sexual violence	In the past 12 months, have you been forced to have sex when you did not want to? By forced, I mean physically forced or verbally threatened. By sex, I mean any sexual contact.	Yes or no
Psychological distress	During the past 30 days, how often did you feel nervous? During the past 30 days, how often did you feel hopeless? During the past 30 days, how often did you feel restless or fidgety? During the past 30 days, how often did you feel so sad or depressed that nothing could cheer you up? During the past 30 days, how often did you feel that everything was an effort? During the past 30 days, how often did you feel down on yourself, no good or worthless?	Experienced psychological distress (had a composite score of 13–24) or did not experience psychological distress (had a composite score below 13)
**Structural syndemic condition**		
Exchange sex	In the past 12 months, have you received money or drugs in exchange for sex?	Yes or no
Homelessness	In the past 12 months, that is, since [interview month] of last year, have you been homeless at any time? By homeless, I mean you were living on the street, in a shelter, in a single room occupancy hotel (SRO), or in a car.	Yes or no
Incarceration	During the past 12 months, that is, since [interview month] of last year, have you been held in a detention center, jail, or prison for more than 24 hours?	Yes or no
**Overall syndemic score**		
Syndemic score	NA	0–6 reported syndemic conditions (homelessness, incarceration, exchange sex, polydrug use, sexual violence, and psychological distress)
**Outcome variable**		
CAI	In the past 12 months, have you had insertive anal sex without a condom? In the past 12 months, have you had receptive anal sex without a condom?	Yes (reported having insertive or receptive anal sex without a condom) or no (reported not having insertive and receptive anal sex without a condom)

**Abbreviations:** CAI = condomless anal intercourse; NA = not applicable; NHBS = National HIV Behavioral Surveillance.

* Atlanta, GA; Los Angeles, CA; New Orleans, LA; New York City, NY; Philadelphia, PA; San Francisco, CA; and Seattle, WA.

^†^ Determined based on NHBS HIV test results.

^§^ Persons of Hispanic or Latina (Hispanic) origin might be of any race but are categorized as Hispanic; all racial groups are non-Hispanic.

^¶^ Determined based on study project area location.

Structural syndemic conditions included past-year experiencing homelessness, past-year incarceration, and past-year exchange sex. Experiencing homelessness was defined as living on the street, in a shelter, in a single-room occupancy hotel, or in a car at any time during the past 12 months. Incarceration was defined as being held in a detention center, jail, or prison for >24 hours during the past 12 months. Exchange sex was defined as ever having received money or drugs in exchange for sex during the past 12 months. A syndemic score was calculated by summing together the number of structural and psychosocial syndemic conditions reported by each participant (range = 0–6). Covariates (age group, education level, relationship status, health insurance, and NHBS HIV test result) were selected based on their potential to confound the relation between syndemic conditions and CAI (*4*,*5*,*13*,*21*).

### Data Analysis

**FIGURE 1:**
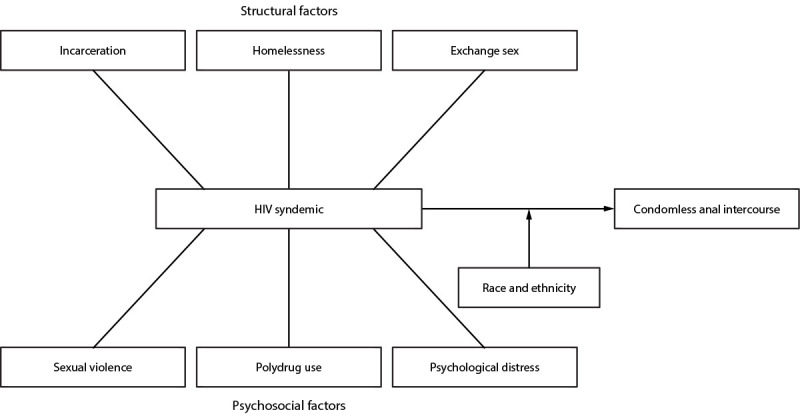
Conceptual model of analysis showing factors contributing to condomless anal intercourse — National HIV Behavioral Surveillance Among Transgender Women, seven urban areas,* United States, 2019–2020. * Atlanta, GA; Los Angeles, CA; New Orleans, LA; New York City, NY; Philadelphia, PA; San Francisco, CA; and Seattle, WA.

This analysis was conducted in four steps using SAS software (version 9.4; SAS Institute). First, descriptive analyses were used to characterize the overall sample and by racial and ethnic groups. Second, the independent associations between syndemic conditions and between each syndemic condition and CAI were estimated and stratified by race and ethnicity. Modified Poisson regression was used to generate adjusted prevalence ratios and 95% CIs for associations between pairs of syndemic conditions and between each syndemic condition and CAI. Third, analyses were conducted to assess whether the strength of the association between syndemic score and CAI differed by race and ethnicity (Figure 1). The effect of syndemic score, race and ethnicity, and interactions among syndemic score and race and ethnicity on CAI were estimated. For significant interaction terms (p<0.05), the effect of the syndemic score on the predicted probability of CAI by racial and ethnic group was estimated and graphed to visualize the relations. Nonsignificant interaction terms were removed from the model. Finally, additive interactions between syndemic conditions on CAI were assessed and stratified by race and ethnicity. The relative excess prevalence owing to interaction (REPI) and 95% CIs were estimated for selected pairs of syndemic conditions (*22*–*27*). Pairs of syndemic conditions were selected based on empirical evidence of potential interactions on CAI among transgender women (*3*–*13*,*28*,*29*). REPI is one of the measures of additive interaction, or the difference of prevalence differences, as a proportion of baseline prevalence (*22*,*24*,*30*). A statistically significant REPI >0 indicates superadditivity and a statistically significant REPI <0 indicates subadditivity (*22*,*24*). Superadditivity indicates that two syndemic conditions produced a larger than expected prevalence of CAI beyond the sum of the independent effects of the conditions (*22*,*24*). Subadditivity indicates that the effects of two syndemic conditions on CAI was lower than the sum of the independent effects of the conditions (*22*,*30*).

The regression analyses were conducted using modified Poisson regression with robust error variance (*31*) and accounted for respondent-driven sampling method by clustering on recruitment chain and adjusting for urban area and network size. Analyses also controlled for covariates (age group, education level, relationship status, health insurance, and NHBS HIV test result).

## Results

The sample comprised 1,348 transgender women (Black = 546, White = 176, and Hispanic = 626) (Table 2). Most participants were aged ≥30 years (68.9%). Nearly half of participants received an HIV-positive test result (43.5%); higher percentages of Black (61.7%) and Hispanic (35.0%) participants received HIV-positive test results compared with White participants (17.0%). The prevalence of syndemic conditions differed by racial and ethnic group. The prevalence of each psychosocial syndemic condition was highest among White participants compared with Black and Hispanic participants (polydrug use: 38.9% [White], 21.1% [Black], and 20.4% [Hispanic]; sexual violence: 18.8% [White], 11.0% [Black], and 16.9% [Hispanic]; psychological distress: 38.3% [White], 22.3% [Black], and 26.5% [Hispanic]).

**TABLE 2:** Number and percentage of sociodemographic characteristics, syndemic conditions, and occurrence of condomless anal intercourse among transgender women, by racial and ethnic group — National HIV Behavioral Surveillance Among Transgender Women,* seven urban areas,^†^ United States, 2019–2020

Characteristic	Black or African American^§^ (n = 546)	White^§^ (n = 176)	Hispanic or Latina^§^ (n = 626)	Total (N = 1,348)
	No. (%)	No. (%)	No. (%)	No. (%)
**Sociodemographic characteristic**				
**Age at interview, yrs^¶^**				
18–24	59 (10.8)	24 (13.7)	78 (12.5)	**161 (12.0)**
25–29	107 (19.6)	39 (22.3)	112 (17.9)	**258 (19.2)**
30–39	165 (30.2)	49 (28.0)	160 (25.6)	**374 (27.8)**
40–49	98 (17.9)	22 (12.6)	135 (21.6)	**255 (18.9)**
≥50	117 (21.4)	41 (23.4)	141 (22.5)	**299 (22.2)**
**Education¶**				
**<**High school	103 (18.9)	14 (8.0)	194 (31.0)	**311 (23.1)**
High school diploma or equivalent	247 (45.3)	52 (29.5)	215 (34.4)	**514 (38.2)**
Some college or technical degree	158 (29.0)	68 (38.6)	164 (26.2)	**390 (29.0)**
College degree or more	37 (6.8)	42 (23.9)	52 (8.3)	**131 (9.7)**
Relationship status^¶,^**				
Single	208 (39.0)	82 (47.4)	274 (44.1)	**564 (42.5)**
Partnered	325 (61.0)	91 (52.6)	347 (55.9)	**763 (57.5)**
**Reported having health insurance**	469 (85.9)	155 (88.1)	497 (79.4)	**1,121 (83.2)**
**NHBS HIV-positive test result^††^**	337 (61.7)	30 (17.0)	219 (35.0)	**586 (43.5)**
**Psychosocial syndemic condition**				
Reported polydrug use past 12 months^¶,§§^	115 (21.1)	68 (38.9)	127 (20.4)	**310 (23.1)**
Experienced sexual violence past 12 months^¶,¶¶^	60 (11.0)	33 (18.8)	105 (16.9)	**198 (14.7)**
Experienced psychological distress past 30 days^¶,^***	122 (22.3)	67 (38.3)	165 (26.5)	**354 (26.3)**
**Structural syndemic condition**				
Reported exchange sex past 12 months^†††^	186 (34.1)	50 (28.4)	222 (35.5)	**458 (34.0)**
Reported homelessness past 12 months^¶,§§§^	238 (43.7)	80 (45.7)	240 (38.3)	**558 (41.5)**
Reported incarceration past 12 months^¶,¶¶¶^	99 (18.2)	23 (13.1)	117 (18.7)	**239 (17.8)**
**Overall syndemic score**				
Syndemic score^¶,^****				
0	157 (29.0)	43 (25.0)	165 (26.7)	**365 (27.4)**
1	149 (27.5)	47 (27.3)	175 (28.4)	**371 (27.9)**
2	111 (20.5)	29 (16.9)	135 (21.9)	**275 (20.7)**
3	70 (12.9)	20 (11.6)	81 (13.1)	**171 (12.8)**
4	44 (8.1)	17 (9.9)	33 (5.3)	**94 (7.1)**
5	9 (1.7)	12 (7.0)	25 (4.1)	**46 (3.5)**
6	2 (0.4)	4 (2.3)	3 (0.5)	**9 (0.7)**
**Outcome variable**				
**Reported CAI past 12 months^††††^**	**288 (52.7)**	**90 (51.1)**	**362 (57.8)**	**740 (54.9)**

**Abbreviations:** CAI = condomless anal intercourse; NHBS = National HIV Behavioral Surveillance.

* N = 1,348 participants had an HIV-negative or HIV-positive NHBS HIV test result; identified as Black or African American, White, or Hispanic or Latina; and had no missing outcome data.

^†^ Atlanta, GA; Los Angeles, CA; New Orleans, LA; New York City, NY; Philadelphia, PA; San Francisco, CA; and Seattle, WA.

^§^ Persons of Hispanic or Latina (Hispanic) origin might be of any race but are categorized as Hispanic; all racial groups are non-Hispanic.

^¶^ Missing: Age: n = 1; education: n = 2; relationship status: n = 21; polydrug use: n = 5; sexual violence: n = 5; psychological distress: n = 4; homelessness: n = 2; incarceration: n = 3; and syndemic score: n = 17.

** Being partnered was defined as having at least one main sexual partner during the past 12 months. A main sexual partner was defined as someone the participant felt committed to above anyone else (e.g., a boyfriend, girlfriend, significant other, or life partner).

^††^ Participants with a reactive rapid NHBS HIV test result confirmed by supplemental rapid or laboratory-based HIV testing were categorized as HIV positive. Participants who self-reported being HIV negative and had a nonreactive rapid NHBS HIV test result were categorized as HIV negative.

^§§^ Polydrug use was defined as having used speedball (combination of heroin and cocaine) or two or more of the following types of drugs via injection or noninjection during the past 12 months: heroin; powder cocaine; crack cocaine; methamphetamine; painkillers (e.g., Oxycontin, Vicodin, morphine, or Percocet); downers (e.g., Klonopin, Valium, Ativan, or Xanax); or poppers or amyl nitrate.

^¶¶^ Sexual violence was defined as having been physically forced or verbally threatened to have sex when they did not want to.

*** Participants with a composite score of 13–24 were categorized as experiencing psychological distress; those with a score <13 were categorized as not experiencing psychological distress.

^†††^ Exchange sex was defined as having received money or drugs in exchange for sex.

^§§§^ Experiencing homelessness was defined as living on the street, in a shelter, in a single room occupancy hotel, or in a car.

^¶¶¶^ Incarceration was defined as being held in a detention center, jail, or prison for >24 hours.

**** An overall syndemic score (0–6) for each participant was calculated by summing reported syndemic conditions (e.g., homelessness, incarceration, exchange sex, polydrug use, sexual violence, and psychological distress).

^††††^ Condomless anal intercourse was defined as having insertive or receptive anal sex without a condom.

For the structural syndemic conditions, the prevalence of exchange sex and incarceration was highest among Black and Hispanic participants compared with White participants (exchange sex: 34.1% [Black], 35.5% [Hispanic], and 28.4% [White]; incarceration: 18.2% [Black] 18.7% [Hispanic], and 13.1% [White]. The prevalence of homelessness was highest among White (45.7%) and Black participants (43.7%) compared with Hispanic participants (38.3%). Twenty-four percent of participants reported three or more syndemic conditions, including 30.8% of White participants, 23.1% of Black participants, and 23.0% of Hispanic participants. Among all participants, 54.9% reported CAI (57.8% of Hispanic participants, 52.7% of Black participants, and 51.1% of White participants).

### Independent Associations Between Syndemic Conditions and Between Syndemic Conditions and CAI

Positive associations between most pairs of syndemic conditions were observed across racial and ethnic groups (Table 3). However, observed associations between syndemic conditions and CAI differed by race and ethnicity. Among Black participants, only exchange sex and polydrug use were independently associated with CAI. For White and Hispanic participants, all associations between syndemic conditions and CAI were significant except for the relation between psychological distress and CAI.

**TABLE 3:** Adjusted prevalence ratios between syndemic conditions and between syndemic conditions and occurrence of condomless anal intercourse among transgender women,* overall and by racial and ethnic group^†^ — National HIV Behavioral Surveillance Among Transgender Women, seven urban areas,^§^ United States, 2019–2020

Characteristic	Syndemic condition						Outcome
	Incarceration	Homelessness	Exchange sex	Polydrug use	Sexual violence	Psychological distress	Condomless anal intercourse
	aPR (95% CI)	aPR (95% CI)	aPR (95% CI)	aPR (95% CI)	aPR (95% CI)	aPR (95% CI)	aPR (95% CI)
**Overall transgender women (N = 1,309)**							
Incarceration	—^¶^	1.85 (1.67–2.06)**	1.29 (1.11– 1.50)**	1.66 (1.28– 2.15)**	1.59 (1.24– 2.05)**	1.15 (0.98– 1.36)	1.21 (1.13– 1.30)**
Homelessness	—	—	1.59 (1.38– 1.82)**	1.97 (1.48– 2.63)**	1.97 (1.55– 2.52)**	1.82 (1.58– 2.11)**	1.15 (1.04– 1.28)**
Exchange sex	—	—	—	2.68 (1.96– 3.66)**	2.94 (2.28– 3.79)**	1.29 (1.08– 1.54)**	1.60 (1.49– 1.71)**
Polydrug use	—	—	—	—	2.24 (1.79– 2.81)**	1.60 (1.36– 1.87)**	1.44 (1.33– 1.56)**
Sexual violence	—	—	—	—	—	1.67 (1.30– 2.14)**	1.39 (1.29– 1.50)**
Psychological distress	—	—	—	—	—	—	1.09 (1.01– 1.19)**
**Black or African American transgender women (n = 530)^††^**							
Incarceration	—	1.51 (1.26– 1.82)**	1.24 (1.00– 1.53)	1.72 (1.30– 2.27)**	1.18 (0.67– 2.08)^§§^	1.68 (1.22– 2.32)**	0.99 (0.81– 1.21)
Homelessness	—	—	1.56 (1.22– 2.00)**	1.72 (1.19– 2.47)**	1.83 (1.00– 3.37)^§§^	1.77 (1.37– 2.28)**	1.10 (0.93– 1.31)
Exchange sex	—	—	—	2.30 (1.48– 3.58)**	3.25 (1.83– 5.79)**^,§§^	1.37 (1.00– 1.88)**	1.60 (1.38– 1.86)**
Polydrug use	—	—	—	—	2.37 (1.37– 4.10)**^,§§^	1.45 (1.03– 2.05)**	1.30 (1.08– 1.56)**
Sexual violence	—	—	—	—	—	1.24 (0.68– 2.25)	1.23 (0.98– 1.56)
Psychological distress	—	—	—	—	—	—	1.02 (0.87– 1.21)
**White transgender women (n = 168)^††^**							
Incarceration	—	1.95 (1.53–2.48)**	2.61 (1.72–3.96)**	2.02 (1.37–2.98)**	2.54 (1.50–4.31)**^,§§^	1.45 (0.93–2.28)	1.96 (1.58–2.44)**
Homelessness	—	—	2.52 (1.27–4.99)**	2.63 (1.52–4.55)**	5.48 (2.08–14.43)**^,§§^	2.12 (1.39–3.22)**	1.62 (1.16–2.25)**
Exchange sex	—	—	—	3.34 (2.20–5.05)**	6.24 (2.95–13.19)**^,§§^	1.28 (0.82–2.01)	1.78 (1.33–2.39)**
Polydrug use	—	—	—	—	2.91 (1.79–4.73)**^,§§^	1.42 (1.02–1.98)**	2.15 (1.64–2.83)**
Sexual violence	—	—	—	—	—	1.49 (1.03–2.14)**	1.72 (1.35–2.20)**
Psychological distress	—	—	—	—	—	—	1.27 (0.96–1.68)
**Hispanic or Latina transgender women (n = 611)^††^**							
Incarceration	—	2.11 (1.78– 2.50)**	1.28 (1.05– 1.56)**	1.65 (1.07– 2.54)**	1.62 (1.12–2.35)**	0.88 (0.68– 1.14)	1.25 (1.13– 1.39)**
Homelessness	—	—	1.53 (1.30– 1.81)**	1.99 (1.35–2.94)**	1.67 (1.22–2.28)**	1.64 (1.35 1.99)**	1.13 (1.00– 1.28)**
Exchange sex	—	—	—	2.87 (2.02– 4.07)**	2.50 (1.64–3.82)**	1.15 (0.85– 1.54)	1.57 (1.43–1.72)**
Polydrug use	—	—	—	—	1.92 (1.44– 2.57)**	1.65 (1.35–2.00)**	1.44 (1.32–1.57)**
Sexual violence	—	—	—	—	—	1.84 (1.45–2.33)**	1.39 (1.27–1.52)**
Psychological distress	—	—	—	—	—	—	1.08 (1.00–1.18)

**Abbreviation:** aPR = adjusted prevalence ratio.

* N = 1,309 participants had an HIV-negative or HIV-positive National HIV Behavioral Surveillance HIV test result; identified as Black or African American, White, or Hispanic or Latina (Hispanic); and had no missing data.

^†^ Models account for respondent-driven sampling method by clustering on recruitment chain and adjusting for urban area and network size. Models also control for age, education level, relationship status, health insurance, and National HIV Behavioral Surveillance HIV test result.

^§^ Atlanta, GA; Los Angeles, CA; New Orleans, LA; New York City, NY; Philadelphia, PA; San Francisco, CA; and Seattle, WA.

^¶^ Dashes indicate no results available.

** aPR statistically significant at p<0.05.

^††^ Persons of Hispanic origin might be of any race but are categorized as Hispanic; all racial groups are non-Hispanic.

^§§^ Models did not control for urban area because models did not converge.

### Association Between Syndemic Score and CAI

**FIGURE 2:**
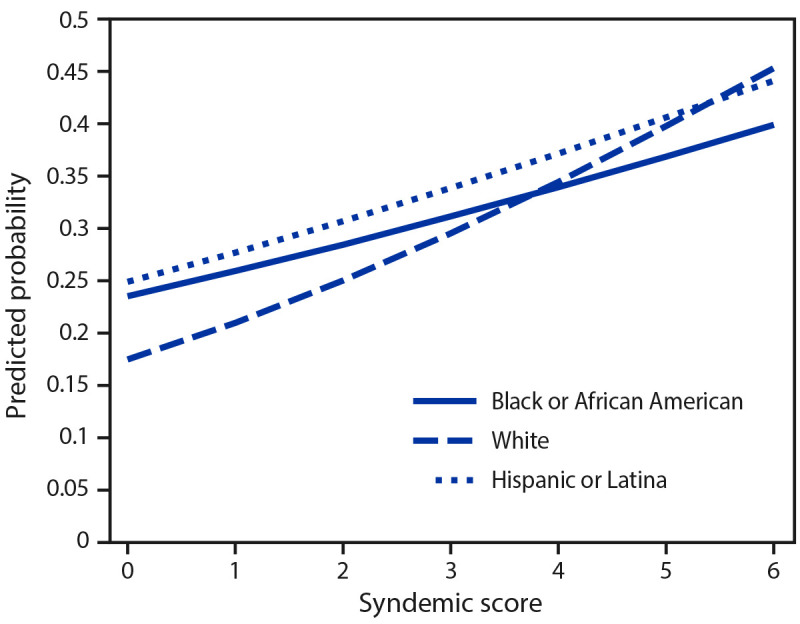
Estimated condomless anal intercourse as a function of syndemic score and race and ethnicity* for Black or African American, White, and Hispanic or Latina transgender women^†^ — National HIV Behavioral Surveillance Among Transgender Women, seven urban areas,^§^ United States, 2019–2020. **Abbreviations:** Black = Black or African American; Hispanic = Hispanic or Latina. * Persons of Hispanic or Latina (Hispanic) origin might be of any race but are categorized as Hispanic; all racial groups are non-Hispanic. ^†^ N = 1,309 participants had an HIV-negative or HIV-positive National HIV Behavioral Surveillance HIV test result; identified as Black, White, or Hispanic; and had no missing data. ^§^ Atlanta, GA; Los Angeles, CA; New Orleans, LA, New York City, NY; Philadelphia, PA; San Francisco, CA, and Seattle, WA.

Reporting more syndemic conditions was significantly associated with reporting CAI for White, Hispanic, and Black participants (Table 4). Both interaction terms for syndemic score by race and ethnicity were statistically significant, illustrating that the association between syndemic score and CAI was significantly stronger for White than Hispanic and Black participants (Table 4) (Figure 2).

**TABLE 4:** Adjusted prevalence ratio estimating the relation between syndemic score and occurrence of condomless anal intercourse,* by racial and ethnic group — National HIV Behavioral Surveillance Among Transgender Women,^†^ seven urban areas,^§^ United States, 2019–2020

Characteristic	CAI aPR (95% CI)
**Syndemic score** ^¶^	1.26 (1.18–1.33)**
**Race and ethnicity^††^**	
Black or African American	1.46 (1.10–1.93)**
White	Ref
Hispanic or Latina	1.57 (1.22–2.00)**
**Syndemic score X Black or African American^¶,††^**	0.91 (0.84–0.98)**
**Syndemic score X Hispanic or Latina^¶,††^**	0.92 (0.86–0.98)**
**Slopes for effect of syndemic score on CAI^††^**	
Black or African American	1.14 (1.09–1.18)**
White	1.26 (1.18–1.33)**
Hispanic or Latina	1.16 (1.13–1.18)**

**Abbreviations:** aPR = adjusted prevalence ratio; CAI = condomless anal intercourse; Ref = referent group.

* Models account for respondent-driven sampling method by clustering on recruitment chain and adjusting for urban area and network size. Models also control for age, education level, relationship status, health insurance, and National HIV Behavioral Surveillance HIV test result.

^†^ N = 1,309 participants had an HIV-negative or HIV-positive National HIV Behavioral Surveillance HIV test result; identified as Black or African American, White, or Hispanic or Latina; and had no missing data.

^§^ Atlanta, GA; Los Angeles, CA; New Orleans, LA; New York City, NY; Philadelphia, PA; San Francisco, CA; and Seattle, WA.

^¶^ An overall syndemic score (0–6) for each participant was calculated by summing reported syndemic conditions (e.g., homelessness, incarceration, exchange sex, polydrug use, sexual violence, and psychological distress).

** aPR statistically significant at p<0.05.

^††^ Persons of Hispanic or Latina (Hispanic) origin might be of any race but are categorized as Hispanic; all racial groups are non-Hispanic.

### REPI Estimates

**FIGURE 3:**
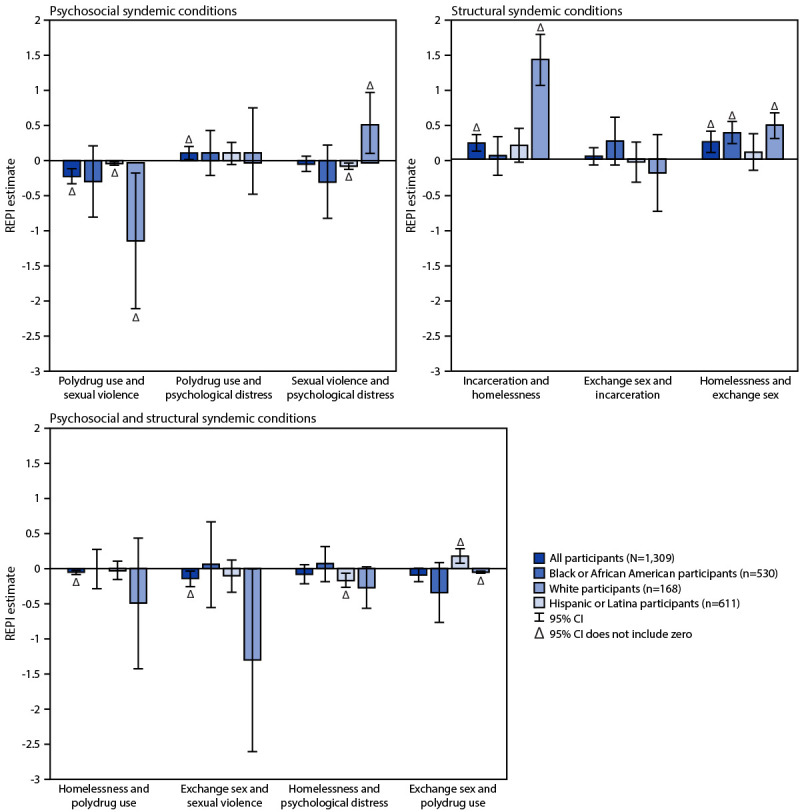
Relative excess prevalence owing to interaction on condomless anal intercourse estimates between syndemic conditions*^,†^ — National HIV Behavioral Surveillance Among Transgender Women,^§^ seven urban areas,^¶^ United States, 2019–2023**^,††^ **Abbreviations:** CAI = condomless anal intercourse; REPI = relative excess prevalence owing to interaction. * Models account for respondent-driven sampling methodology by clustering on recruitment chain and adjusting for urban area. Models also control for age, education level, relationship status, health insurance, and National HIV Behavioral Surveillance HIV test result. ^†^ An REPI estimate >0 indicates superadditivity between syndemic conditions on CAI. A REPI estimate <0 indicates subadditivity effects between syndemic conditions on CAI. ^§^ Persons of Hispanic or Latina (Hispanic) origin might be of any race but are categorized as Hispanic; all racial groups are non-Hispanic. ^¶^ Atlanta, GA; Los Angeles, CA; New Orleans, LA, New York City, NY; Philadelphia, PA; San Francisco, CA, and Seattle, WA. ** REPI estimates with a 95% CI that does not include zero are marked with a triangle (∆). ^††^ N = 1,309 participants had an HIV-negative or HIV-positive NHBS HIV test result; identified as Black, White, or Hispanic; and had no missing data.

REPI estimates differed by race and ethnicity and by pairs of syndemic conditions (Figure 3). The directionality of REPI estimates often differed by racial and ethnic group, indicating that interactions between the same pair of syndemic conditions might be positive or superadditive for certain racial and ethnic groups and negative or subadditive for others. For REPI estimates between psychosocial syndemic conditions, there were significant REPI estimates for subadditive interactions between polydrug use and sexual violence on CAI prevalence among White (REPI = −1.11; 95% CI = −2.08 to −0.14) and Hispanic participants (REPI = −0.04; 95% CI = −0.07 to −0.01) and between sexual violence and psychological distress on CAI prevalence among Hispanic participants (REPI = −0.08; 95% CI = −0.13 to −0.03). A superadditive interaction was observed between sexual violence and psychological distress on CAI prevalence for White participants (REPI = 0.54; 95% CI = 0.10–0.99). For the significant REPI estimates between structural syndemic conditions, superadditive interactions were observed for incarceration and homelessness on CAI prevalence among White participants (REPI = 1.44; 95% CI = 1.06–1.81) and homelessness and exchange sex on CAI prevalence among Black (REPI = 0.38; 95% CI = 0.22–0.55) and White participants (REPI = 0.49; 95% CI = 0.29–0.68). For the REPI estimates between selected pairs of structural and psychosocial syndemic conditions, the only significant positive REPI estimate was between exchange sex and polydrug use among Hispanic participants (REPI = 0.18; 95% CI = 0.07–0.29). Significant negative REPI estimates were found between homelessness and psychological distress on CAI prevalence among Hispanic participants (REPI = −0.17; 95% CI = −0.27 to −0.06) and between exchange sex and polydrug use among White participants (REPI = −0.05; 95% CI = −0.07 to −0.03).

## Discussion

In this analysis, syndemic conditions and CAI were prevalent among transgender women. Independent associations between syndemic conditions and between syndemic conditions and CAI were observed, demonstrating that HIV prevention efforts for transgender women should address structural and psychosocial syndemic conditions (*10*,*11*,*32*). Further, reporting more syndemic conditions was associated with increased CAI prevalence across racial and ethnic groups. Findings are consistent with other studies examining relations between co-occurring syndemic conditions and behaviors associated with HIV transmission among transgender women (*3*–*6*).

This analysis adds to the literature by testing for additive interactions between selected combinations of structural and psychosocial syndemic conditions on CAI. Similar analytic approaches have been used in research with men who have sex with men and help identify specific combinations of syndemic conditions that result in increased likelihood of behaviors associated with HIV transmission and help develop tailored intervention responses (*25*,*27*,*33*,*34*). In this analysis, limited significant superadditive interactions were found, although the majority were between structural syndemic conditions (e.g., superadditive interactions between experiencing homelessness and exchange sex on CAI prevalence among Black and White participants). These results underscore the importance of prioritizing HIV prevention interventions that address social determinants of health (e.g., housing and poverty) (*32*,*35*). Notably, the same combinations of syndemic conditions often resulted in a superadditive interaction for one racial and ethnic group and a subadditive interaction for another racial and ethnic group. For example, a subadditive interaction between sexual violence and psychological distress was found among Hispanic participants, and a superadditive interaction was found among White participants. These differences in interaction results demonstrate the need to tailor syndemic interventions to racial and ethnic groups. Additional research is also needed to explore why interactions might differ across racial and ethnic groups.

Prevalence estimates for syndemic conditions differed by race and ethnicity. Psychosocial syndemic conditions were reported most frequently by White participants, which might be explained in multiple ways. First, polydrug use might be higher among White participants because the opioid epidemic disproportionately affects White persons (*36*). More research is needed to improve understanding of racial and ethnic differences in substance use among transgender women (*37*). Second, John Henryism might explain the lower levels of reported psychological distress among Black participants (*38*,*39*). John Henryism is a high-effort, active coping style often used by Black persons to deal with psychosocial and environmental stressors (*38*,*39*). Studies have found associations between John Henryism and increased physical health problems (e.g., hypertension) among Black residents of the United States, especially Black men (*38*,*40*). Certain studies have also found that John Henryism is associated with reduced reporting of mental health problems, although additional research is needed (*41*–*43*). Alternatively, another mental health measure (e.g., depressive symptoms) might better capture mental health problems affecting this population than does psychological distress. Other studies have had mixed results and found Black gender minority participants are at lower or equal risk for depression compared with White gender minority participants (*44*,*45*).

Racial and ethnic differences also were observed in reported structural syndemic conditions. Black and Hispanic participants reported higher levels of exchange sex and incarceration than White participants. The higher prevalence of exchange sex might be a result of more severe economic marginalization because of racial and ethnic discrimination (*13*), and the higher prevalence of incarceration is likely because of the disproportionate impact of mass incarceration on Black and Hispanic populations (*46*,*47*). However, structural and psychosocial syndemic conditions were prevalent across racial and ethnic groups, underscoring the importance of addressing syndemic conditions for all transgender women.

Findings differed by racial and ethnic group, highlighting the importance of assessing racial and ethnic differences in HIV prevention research among transgender women (*48*). Syndemic theory emphasizes that disparities in health outcomes or interactions between health outcomes are produced by social or environmental factors (*14*,*15*). Future syndemics research should consider including racial and ethnic discrimination measures and apply an intersectional framework to improve understanding on how social and environmental factors produce racial and ethnic disparities in syndemic conditions and behaviors associated with HIV transmission among transgender women (*44*,*49*–*52*). In addition, testing protective factors (e.g., resilience and social support) as effect modifiers might help explain racial and ethnic differences in associations between syndemic conditions and behaviors associated with HIV transmission (*6*).

## Limitations

General limitations for NHBS-Trans are available in the overview and methodology report of this supplement (*17*). The findings in this report are subject to at least six additional limitations. First, temporality between syndemic conditions and between the syndemic conditions and CAI could not be assessed because of the cross-sectional study design and overlapping recall periods for measures. Nevertheless, both structural and psychosocial syndemic conditions were included in the analysis, which allowed for testing of additive interactions between structural syndemic conditions, psychosocial syndemic conditions, and structural and psychosocial syndemic conditions on CAI prevalence. Second, the sample is not representative of transgender women residing outside of the seven urban areas. Because transgender women are hard to reach, the data might not be representative of all transgender women residing in the seven urban areas. However, data were collected from multiple diverse urban areas using a robust, standardized surveillance system (*2*). Third, the sample size differed by racial and ethnic group and was most limited among White participants, which likely influenced the precision of parameter estimates and might have reduced power to detect associations. Nonetheless, Black and Hispanic populations disproportionately affected by HIV were over sampled, allowing for a stratified analysis to examine racial and ethnic differences in associations between syndemic conditions and CAI (*2*). Fourth, participants may have been at low or minimal risk of acquiring or transmitting HIV through CAI if they were taking preexposure prophylaxis or HIV treatment medication as prescribed; whether participants with HIV had an undetectable viral load or participants without HIV had full protection from taking preexposure prophylaxis every time they had CAI could not be determined. Fifth, multiple comparisons were not adjusted, increasing the likelihood of type I errors when assessing independent associations between syndemic conditions and between syndemic conditions and CAI. Finally, all measures except for NHBS HIV test result were self-reported, which might be subject to social desirability bias and result in underreporting of syndemic conditions and CAI (*53*–*55*).

## Conclusions

Because of the high prevalence of syndemic conditions and CAI, culturally sensitive HIV prevention and behavioral, biomedical, and structural interventions for transgender women are urgently needed (*10*,*11*,*32*). HIV behavioral interventions addressing risks associated with certain sexual behaviors, mental health symptoms, and substance use for transgender women have reduced behaviors associated with HIV transmission (*56*–*60*). Although limited, behavioral interventions designed to address psychosocial and structural syndemic conditions (e.g., homelessness, legal employment and income, and mental health symptoms among transgender women) also have indicated promising reductions in behaviors associated with HIV transmission (*35*,*61*–*63*). Findings indicated differences in prevalence of syndemic conditions and interactions between syndemic conditions on CAI by racial and ethnic group, suggesting that syndemic-focused interventions for transgender women should be tailored to racial and ethnic groups. Results indicate that syndemic-focused interventions for Black transgender women should address the intersection between experiencing homelessness and exchange sex; those for Hispanic transgender women should address the intersection between exchange sex and polydrug use; and those for White transgender women should address the intersections between sexual violence and psychological distress, incarceration and experiencing homelessness, and experiencing homelessness and exchange sex. Culturally tailored syndemic-focused interventions that offer comprehensive services addressing social and structural barriers to status-neutral HIV services might be effective (*64*,*65*). For example, interventions designed for transgender women of color with HIV infection have increased engagement in HIV care by offering patient navigation or case management, housing and employment assistance, mental health services, and substance use services (*65*). Although HIV behavioral interventions have proven efficacy among transgender women, multilevel interventions are also critical to reduce gender-identity–related and racial- and ethnic–related stigma and discrimination and increase access to pre-exposure prophylaxis, HIV treatment, and gender-affirming medical care (*32*,*66*).

## Footnotes

* 45 C.F.R. part 46, 21 C.F.R. part 56; 42 U.S.C. Sect. 241(d); 5 U.S.C. Sect. 552a; 44 U.S.C. Sect. 3501 et seq.

## References

[1] Becasen JS, Denard CL, Mullins MM, Higa DH, Sipe TA. Estimating the prevalence of HIV and sexual behaviors among the U.S. transgender population: a systematic review and meta-analysis, 2006–2017. Am J Public Health 2019;109:e1–8. 10.2105/AJPH.2018.304727 30496000 PMC6301428

[2] CDC. HIV infection, risk, prevention, and testing behaviors among transgender women—National HIV Behavioral Surveillance, 7 U.S. cities, 2019–2020. Atlanta, GA: US Department of Health and Human Services, CDC; 2021.

[3] Brennan J, Kuhns LM, Johnson AK, Belzer M, Wilson EC, Garofalo R; Adolescent Medicine Trials Network for HIV/AIDS Interventions. Syndemic theory and HIV-related risk among young transgender women: the role of multiple, co-occurring health problems and social marginalization. Am J Public Health 2012;102:1751–7. 10.2105/AJPH.2011.300433 22873480 PMC3416048

[4] Mimiaga MJ, Hughto JMW, Biello KB, Longitudinal analysis of syndemic psychosocial problems predicting HIV risk behavior among a multicity prospective cohort of sexually active young transgender women in the United States. J Acquir Immune Defic Syndr 2019;81:184–92. 10.1097/QAI.0000000000002009 30839380 PMC6522320

[5] Parsons JT, Antebi-Gruszka N, Millar BM, Cain D, Gurung S. Syndemic conditions, HIV transmission risk behavior, and transactional sex among transgender women. AIDS Behav 2018;22:2056–67. 10.1007/s10461-018-2100-y 29589136 PMC6021215

[6] Teixeira da Silva D, Bouris A, Voisin D, Hotton A, Brewer R, Schneider J. Social networks moderate the syndemic effect of psychosocial and structural factors on HIV risk among young black transgender women and men who have sex with men. AIDS Behav 2020;24:192–205. 10.1007/s10461-019-02575-9 31289985 PMC7263264

[7] Wilson EC, Chen YH, Arayasirikul S, Differential HIV risk for racial/ethnic minority trans*female youths and socioeconomic disparities in housing, residential stability, and education. Am J Public Health 2015;105(Suppl 3):e41–7. 10.2105/AJPH.2014.302443 25905826 PMC4455493

[8] Eastwood EA, Nace AJ, Hirshfield S, Birnbaum JM. Young transgender women of color: homelessness, poverty, childhood sexual abuse and implications for HIV care. AIDS Behav 2021;25(Suppl 1):96–106. 10.1007/s10461-019-02753-9 31865517

[9] Gilbert L, Raj A, Hien D, Stockman J, Terlikbayeva A, Wyatt G. Targeting the SAVA (substance abuse, violence, and AIDS) syndemic among women and girls: a global review of epidemiology and integrated interventions. J Acquir Immune Defic Syndr 2015;69(Suppl 2):S118–27. 10.1097/QAI.0000000000000626 25978478 PMC4751344

[10] Poteat T, Reisner SL, Radix A. HIV epidemics among transgender women. Curr Opin HIV AIDS 2014;9:168–73. 10.1097/COH.0000000000000030 24322537 PMC5947322

[11] Poteat T, Scheim A, Xavier J, Reisner S, Baral S. Global epidemiology of HIV infection and related syndemics affecting transgender people. J Acquir Immune Defic Syndr 2016;72(Suppl 3):S210–9. 10.1097/QAI.0000000000001087 27429185 PMC4969059

[12] Reisner SL, Poteat T, Keatley J, Global health burden and needs of transgender populations: a review. Lancet 2016;388:412–36. 10.1016/S0140-6736(16)00684-X 27323919 PMC7035595

[13] Arrington-Sanders R, Alvarenga A, Galai N, Social determinants of transactional sex in a sample of young Black and Latinx sexual minority cisgender men and transgender women. J Adolesc Health 2022;70:275–81. 10.1016/j.jadohealth.2021.08.002 34580030 PMC8915132

[14] Singer M. AIDS and the health crisis of the U.S. urban poor; the perspective of critical medical anthropology. Soc Sci Med 1994;39:931–48. 10.1016/0277-9536(94)90205-4 7992126

[15] Singer MC, Erickson PI, Badiane L, Syndemics, sex and the city: understanding sexually transmitted diseases in social and cultural context. Soc Sci Med 2006;63:2010–21. 10.1016/j.socscimed.2006.05.012 16782250 PMC7131051

[16] Poteat T, Malik M, Wirtz AL, Cooney EE, Reisner S. Understanding HIV risk and vulnerability among cisgender men with transgender partners. Lancet HIV 2020;7:e201–8. 10.1016/S2352-3018(19)30346-7 32032535

[17] Kanny D, Lee K, Olansky E, Overview and methodology of the National HIV Behavioral Surveillance among Transgender Women—seven urban areas, United States, 2019–2020. In: National HIV Behavioral Surveillance Among Transgender Women—seven urban areas, United States, 2019–2020. MMWR Suppl 2024;73(No. Suppl-1):1–8. 10.15585/mmwr.su7301a1 PMC10826683 38284875

[18] Kessler RC, Andrews G, Colpe LJ, Short screening scales to monitor population prevalences and trends in non-specific psychological distress. Psychol Med 2002;32:959–76. 10.1017/S0033291702006074 12214795

[19] Kessler RC, Barker PR, Colpe LJ, Screening for serious mental illness in the general population. Arch Gen Psychiatry 2003;60:184–9. 10.1001/archpsyc.60.2.184 12578436

[20] Almazan AN, Keuroghlian AS. Association between gender-affirming surgeries and mental health outcomes. JAMA Surg 2021;156:611–8. 10.1001/jamasurg.2021.0952 33909023 PMC8082431

[21] Kisler KA, Fletcher JB, Fehrenbacher AE, Reback CJ. Age is associated with HIV sexual risk behaviors among trans women in Los Angeles County. AIDS Educ Prev 2021;33:483–94. 10.1521/aeap.2021.33.6.483 34874757 PMC10445543

[22] VanderWeele TJ, Knol MJ. A tutorial on interaction. Epidemiol Methods 2014;3:33–72. 10.1515/em-2013-0005

[23] Richardson DB, Kaufman JS. Estimation of the relative excess risk due to interaction and associated confidence bounds. Am J Epidemiol 2009;169:756–60. 10.1093/aje/kwn411 19211620 PMC3139969

[24] Hosmer DW, Lemeshow S. Confidence interval estimation of interaction. Epidemiology 1992;3:452–6. 10.1097/00001648-199209000-00012 1391139

[25] Jiang H, Li J, Tan Z, Syndemic factors and HIV risk among men who have sex with men in Guangzhou, China: evidence from synergy and moderated analyses. Arch Sex Behav 2020;49:311–20. 10.1007/s10508-019-01488-x 31617111

[26] Lee K, Hutton HE, Lesko CR, Associations of drug use, violence, and depressive symptoms with sexual risk behaviors among women with alcohol misuse. Womens Health Issues 2018;28:367–74. 10.1016/j.whi.2018.04.004 29784276 PMC6592607

[27] Tomori C, McFall AM, Solomon SS, Is there synergy in syndemics? Psychosocial conditions and sexual risk among men who have sex with men in India. Soc Sci Med 2018;206:110–6. 10.1016/j.socscimed.2018.03.032 29615297 PMC5955386

[28] Anderson-Carpenter KD, Fletcher JB, Reback CJ. Associations between methamphetamine use, housing status, and incarceration rates among men who have sex with men and transgender women. J Drug Issues 2017;47:383–95. 10.1177/0022042617696917 28670005 PMC5485860

[29] Fletcher JB, Kisler KA, Reback CJ. Housing status and HIV risk behaviors among transgender women in Los Angeles. Arch Sex Behav 2014;43:1651–61. 10.1007/s10508-014-0368-1 25190499 PMC4214608

[30] Rothman KJ, Greenland S, Lash TL. Modern epidemiology. 3rd ed. Philadelphia, PA: Lippincott Williams & Wilkins; 2008.

[31] Zou G. A modified poisson regression approach to prospective studies with binary data. Am J Epidemiol 2004;159:702–6. 10.1093/aje/kwh090 15033648

[32] Poteat T, Malik M, Scheim A, Elliott A. HIV prevention among transgender populations: knowledge gaps and evidence for action. Curr HIV/AIDS Rep 2017;14:141–52. 10.1007/s11904-017-0360-1 28752285 PMC5896563

[33] Tsai AC, Burns BF. Syndemics of psychosocial problems and HIV risk: a systematic review of empirical tests of the disease interaction concept. Soc Sci Med 2015;139:26–35. 10.1016/j.socscimed.2015.06.024 26150065 PMC4519429

[34] Tsai AC, Venkataramani AS. Syndemics and health disparities: a methodological note. AIDS Behav 2016;20:423–30. 10.1007/s10461-015-1260-2 26662266 PMC4755906

[35] Hill BJ, Motley DN, Rosentel K, Employment as HIV prevention: an employment support intervention for adolescent men who have sex with men and adolescent transgender women of color. J Acquir Immune Defic Syndr 2022;91:31–8. 10.1097/QAI.0000000000003020 35551157 PMC9377485

[36] Restar AJ, Jin H, Ogunbajo A, Prevalence and risk factors of nonmedical prescription opioid use among transgender girls and young women. JAMA Netw Open 2020;3:e201015. 10.1001/jamanetworkopen.2020.1015 32176305 PMC7076341

[37] Ruppert R, Kattari SK, Sussman S. Review: prevalence of addictions among transgender and gender diverse subgroups. Int J Environ Res Public Health 2021;18:8843. 10.3390/ijerph18168843 34444595 PMC8393320

[38] Bennett GG, Merritt MM, Sollers JJ III, Stress, coping, and health outcomes among African-Americans: a review of the John Henryism hypothesis. Psychol Health 2004;19:369–83. 10.1080/0887044042000193505

[39] James SA. John Henryism and the health of African-Americans. Cult Med Psychiatry 1994;18:163–82. 10.1007/BF01379448 7924399

[40] Felix AS, Shisler R, Nolan TS, High-effort coping and cardiovascular disease among women: a systematic review of the John Henryism hypothesis. J Urban Health 2019;96(Suppl 1):12–22. 10.1007/s11524-018-00333-1 30506136 PMC6430283

[41] Robinson MN, Thomas Tobin CS. Is John Henryism a health risk or resource? Exploring the role of culturally relevant coping for physical and mental health among Black Americans. J Health Soc Behav 2021;62:136–51. 10.1177/00221465211009142 34100655 PMC8370445

[42] Bronder EC, Speight SL, Witherspoon KM, Thomas AJ. John Henryism, depression, and perceived social support in Black women. J Black Psychol 2014;40:115–37. 10.1177/0095798412474466

[43] Kiecolt KJ, Hughes M, Keith VM. Can a high sense of control and John Henryism be bad for mental health? Sociol Q 2009;50:693–714. 10.1111/j.1533-8525.2009.01152.x

[44] Lett E, Dowshen NL, Baker KE. Intersectionality and health inequities for gender minority Blacks in the U.S. Am J Prev Med 2020;59:639–47. 10.1016/j.amepre.2020.04.013 32792281 PMC7577994

[45] Brown GR, Jones KT. Racial health disparities in a cohort of 5,135 transgender veterans. J Racial Ethn Health Disparities 2014;1:257–66. 10.1007/s40615-014-0032-4

[46] Reisner SL, Bailey Z, Sevelius J. Racial/ethnic disparities in history of incarceration, experiences of victimization, and associated health indicators among transgender women in the U.S. Women Health 2014;54:750–67. 10.1080/03630242.2014.932891 25190135 PMC5441521

[47] Wildeman C, Wang EA. Mass incarceration, public health, and widening inequality in the USA. Lancet 2017;389:1464–74. 10.1016/S0140-6736(17)30259-3 28402828

[48] del Río-González AM, Lameiras-Fernández M, Modrakovic D, Global scoping review of HIV prevention research with transgender people: transcending from trans-subsumed to trans-centred research. J Int AIDS Soc 2021;24:e25786. 10.1002/jia2.25786 34473421 PMC8412127

[49] Biello KB, Hughto JMW. Measuring intersectional stigma among racially and ethnically diverse transgender women: challenges and opportunities. Am J Public Health 2021;111:344–6. 10.2105/AJPH.2020.306141 33566645 PMC7893348

[50] Wesson P, Vittinghoff E, Turner C, Arayasirikul S, McFarland W, Wilson E. Intercategorical and intracategorical experiences of discrimination and HIV prevalence among transgender women in San Francisco, CA: a quantitative intersectionality analysis. Am J Public Health 2021;111:446–56. 10.2105/AJPH.2020.306055 33476238 PMC7893335

[51] Wesp LM, Malcoe LH, Elliott A, Poteat T. Intersectionality research for transgender health justice: a theory-driven conceptual framework for structural analysis of transgender health inequities. Transgend Health 2019;4:287–96. 10.1089/trgh.2019.0039 31663035 PMC6818474

[52] Smith LR, Patel VV, Tsai AC, Integrating intersectional and syndemic frameworks for ending the U.S. HIV epidemic. Am J Public Health 2022;112(S4):S340–3. 10.2105/AJPH.2021.306634 35763739 PMC9241475

[53] Latkin CA, Edwards C, Davey-Rothwell MA, Tobin KE. The relationship between social desirability bias and self-reports of health, substance use, and social network factors among urban substance users in Baltimore, Maryland. Addict Behav 2017;73:133–6. 10.1016/j.addbeh.2017.05.005 28511097 PMC5519338

[54] Perinelli E, Gremigni P. Use of social desirability scales in clinical psychology: a systematic review. J Clin Psychol 2016;72:534–51. 10.1002/jclp.22284 26970350

[55] Rao A, Tobin K, Davey-Rothwell M, Latkin CA. Social desirability bias and prevalence of sexual HIV risk behaviors among people who use drugs in Baltimore, Maryland: implications for identifying individuals prone to underreporting sexual risk behaviors. AIDS Behav 2017;21:2207–14. 10.1007/s10461-017-1792-8 28509997 PMC5521816

[56] Bockting WO, Robinson BE, Forberg J, Scheltema K. Evaluation of a sexual health approach to reducing HIV/STD risk in the transgender community. AIDS Care 2005;17:289–303. 10.1080/09540120412331299825 15832877

[57] Garofalo R, Johnson AK, Kuhns LM, Cotten C, Joseph H, Margolis A. Life skills: evaluation of a theory-driven behavioral HIV prevention intervention for young transgender women. J Urban Health 2012;89:419–31. 10.1007/s11524-011-9638-6 22223033 PMC3368050

[58] Nemoto T, Operario D, Keatley J, Nguyen H, Sugano E. Promoting health for transgender women: Transgender Resources and Neighborhood Space (TRANS) program in San Francisco. Am J Public Health 2005;95:382–4. 10.2105/AJPH.2004.040501 15727962 PMC1449187

[59] Taylor RD, Bimbi DS, Joseph HA, Margolis AD, Parsons JT. Girlfriends: evaluation of an HIV-risk reduction intervention for adult transgender women. AIDS Educ Prev 2011;23:469–78. 10.1521/aeap.2011.23.5.469 22010810

[60] Garofalo R, Kuhns LM, Reisner SL, Biello K, Mimiaga MJ. Efficacy of an empowerment-based, group-delivered HIV prevention intervention for young transgender women: the Project LifeSkills randomized clinical trial. JAMA Pediatr 2018;172:916–23. 10.1001/jamapediatrics.2018.1799 30105381 PMC6233762

[61] Reback CJ, Clark K, Fletcher JB. TransAction: a homegrown, theory-based, HIV risk reduction intervention for transgender women experiencing multiple health disparities. Sex Res Soc Policy 2019;16:408–18. 10.1007/s13178-018-0356-7 33133300 PMC7597668

[62] Reback CJ, Shoptaw S, Downing MJ. Prevention case management improves socioeconomic standing and reduces symptoms of psychological and emotional distress among transgender women. AIDS Care 2012;24:1136–44. 10.1080/09540121.2012.687817 22670654 PMC5832491

[63] Martinez O, Lopez N, Woodard T, Rodriguez-Madera S, Icard L. Transhealth Information Project: a peer-led HIV prevention intervention to promote HIV protection for individuals of transgender experience. Health Soc Work 2019;44:104–12. 10.1093/hsw/hlz008 30855670 PMC6642448

[64] Goldhammer H, Marc LG, Psihopaidas D, HIV care continuum interventions for transgender women: a topical review. Public Health Rep 2023;138:19–30. 10.1177/00333549211065517 35060802 PMC9730173

[65] Rebchook GM, Chakravarty D, Xavier JM, ; SPNS Transgender Women of Color Study Group. An evaluation of nine culturally tailored interventions designed to enhance engagement in HIV care among transgender women of colour in the United States. J Int AIDS Soc 2022;25(Suppl 5):e25991. 10.1002/jia2.25991 36225153 PMC9557010

[66] Gamarel KE, Rebchook G, McCree BM, The ethical imperative to reduce HIV stigma through community-engaged, status-neutral interventions designed with and for transgender women of colour in the United States. J Int AIDS Soc 2022;25(Suppl 1):e25907. 10.1002/jia2.25907 35818894 PMC9274348

